# Appendicular Schistosomiasis Presenting as Peritonitis in the Eastern Province of Saudi Arabia: A Case Report

**DOI:** 10.7759/cureus.83377

**Published:** 2025-05-02

**Authors:** Wafa M Almuzayil, Mohmmed A AlHewishel, Husain A Alsaffar, Abdulrahman A Alarfaj, Fahad S AlMutairi, Nawaf Abdulrahim

**Affiliations:** 1 General Surgery, Dammam Medical Complex, Dammam, SAU; 2 General Surgery, Imam Abdulrahman Bin Faisal University, Dammam, SAU; 3 Surgery, King Fahad Specialist Hospital, Dammam, SAU

**Keywords:** appendicitis, case report, peritonitis, praziquantel, saudi arabia, s: schistosomiasis

## Abstract

We report the case of a 17-year-old Yemeni male with no prior medical or surgical history who presented with generalized abdominal pain, nausea, vomiting, anorexia, and diarrhea. Initial clinical and radiological evaluations suggested diffuse peritonitis, with findings consistent with appendicitis. The patient underwent an open laparotomy and appendectomy, which revealed a perforated appendix with purulent contamination. Histopathological examination confirmed schistosomal ova in the appendix. Postoperative complications included a fluid collection, which was managed with targeted antibiotics. The patient was also treated with praziquantel and eventually recovered well. This case highlights the rare occurrence of schistosomiasis presenting as appendicular peritonitis and emphasizes the importance of considering parasitic infections in acute abdominal presentations, particularly in endemic regions.

## Introduction

Schistosomiasis is a parasitic disease caused by trematode worms of the genus *Schistosoma*, affecting over 200 million people globally, primarily in tropical and subtropical regions [1]. While the disease commonly involves the urinary and gastrointestinal tracts, leading to conditions such as hematuria, diarrhea, and hepatic fibrosis, appendiceal involvement is relatively rare [2]. The prevalence of schistosomal appendicitis varies geographically, with studies reporting rates ranging from 1.31% to 3.2% in endemic areas [3]. However, the progression of schistosomal appendicitis to generalized peritonitis is exceedingly uncommon [3,4].

The pathogenesis of schistosomal appendicitis involves granulomatous inflammation and fibrosis caused by the deposition of *Schistosoma* eggs, leading to luminal obstruction and secondary bacterial infection. *Schistosoma mansoni* and *Schistosoma japonicum* are most frequently associated with intestinal and hepatosplenic disease [5]. Histopathological examination remains essential for diagnosis, as the clinical presentation mimics that of non-parasitic acute appendicitis [6]. Some studies have reported incidental findings of schistosomal ova in appendectomy specimens, further supporting the need for routine pathological evaluation in endemic areas [7].

This case report highlights a rare presentation of appendicular schistosomiasis leading to peritonitis. It underscores the importance of considering parasitic infections as a differential diagnosis in patients from endemic regions or with relevant travel histories [8].

## Case presentation

A 17-year-old Yemeni male with no significant medical or surgical history presented to the emergency department with a three-day history of generalized abdominal pain, nausea, vomiting, anorexia, and watery diarrhea without blood or mucus. The patient denied any urinary symptoms, jaundice, or changes in the color of his urine or stool. He reported recent consumption of food from an external source but did not mention any travel outside Yemen.

On examination, the patient appeared dehydrated, with a temperature of 38.5°C, blood pressure of 105/63 mmHg, heart rate of 77 bpm, and oxygen saturation of 97% on room air. Abdominal examination revealed generalized tenderness with guarding. Laboratory investigations showed an elevated white blood cell count of 11.05 x 10⁹/L and a lactate of 3 mmol/L. Liver and renal function tests were normal (Table 1). Based on the clinical presentation and laboratory findings, the patient’s Alvarado Score was calculated to be 9 out of 10, indicating a high probability of acute appendicitis. The score components included anorexia, nausea and vomiting, right lower quadrant tenderness, guarding, fever (38.5°C), leukocytosis (white blood cells (WBC) 11.05 × 10⁹/L), and neutrophilia (94%). This clinical scoring further supported the decision to proceed with surgical intervention.

**Table 1:** Renal and liver function test results upon admission. GFR: Glomerular filtration rate, AST (SGOT): Aspartate transaminase (serum glutamic-oxaloacetic transaminase), ALT (SGPT): Alanine aminotransferase (serum glutamic-pyruvic transaminase)

Parameter	Result	Reference Range	Interpretation
Blood Urea Nitrogen (BUN)	12 mg/dL	7 – 20 mg/dL	Normal
Creatinine	0.9 mg/dL	0.6 – 1.3 mg/dL	Normal
Estimated GFR	100 mL/min/1.73m²	> 90 mL/min/1.73m²	Normal
AST (SGOT)	22 U/L	10 – 40 U/L	Normal
ALT (SGPT)	25 U/L	7 – 56 U/L	Normal
Alkaline Phosphatase (ALP)	88 U/L	40 – 129 U/L	Normal
Total Bilirubin	0.7 mg/dL	0.1 – 1.2 mg/dL	Normal
Albumin	4.2 g/dL	3.5 – 5.0 g/dL	Normal

**Figure 1:**
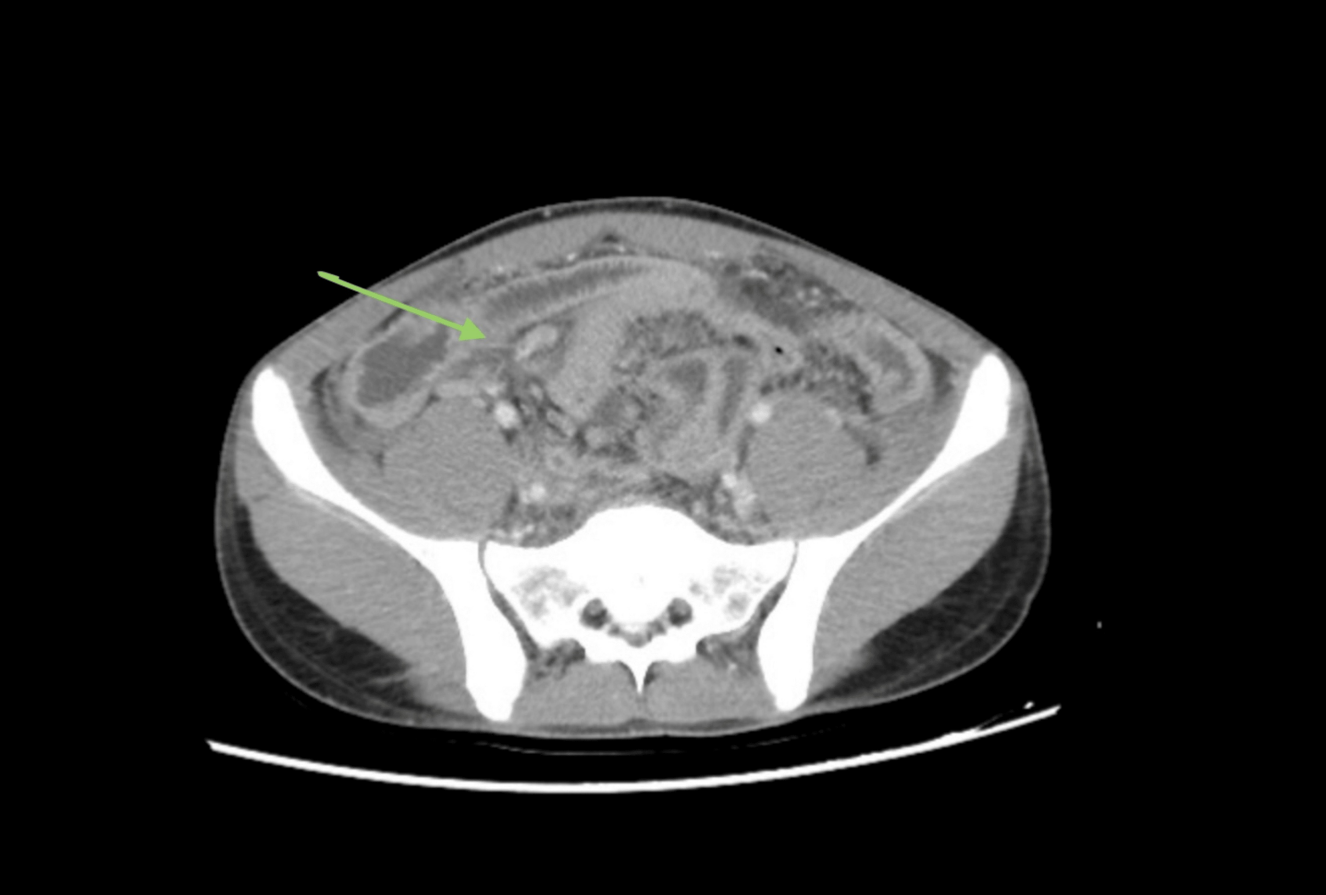
CT image demonstrating a distended, fluid-filled appendix (9 mm in diameter) with surrounding fat stranding—features consistent with acute appendicitis.

A contrast-enhanced abdominal CT scan revealed free fluid in multiple compartments (peri-hepatic, peri-splenic, and pelvic), thickening of the peritoneal reflection, and diffuse mesenteric fat stranding, suggestive of peritonitis. The appendix appeared enlarged, with a diameter of 9 mm and surrounding fat stranding, consistent with appendicitis (Figure 1). 

Surgical intervention and postoperative course

The patient was admitted to the general surgery service and started on intravenous fluids, antibiotics (Tazocin), analgesia, and a proton pump inhibitor. He underwent an open laparotomy, which revealed significant serous and purulent fluid throughout the peritoneal cavity along with dilated small bowel loops. The appendix, located in the right lower quadrant, was perforated and surrounded by purulent material. After ligating and dividing the mesoappendix, the appendix was excised, and the appendiceal stump was oversewn. The specimen was sent for histopathological examination, and the peritoneal cavity was thoroughly irrigated. An 18-French drain was placed in the left lower quadrant prior to wound closure.

**Figure 2:**
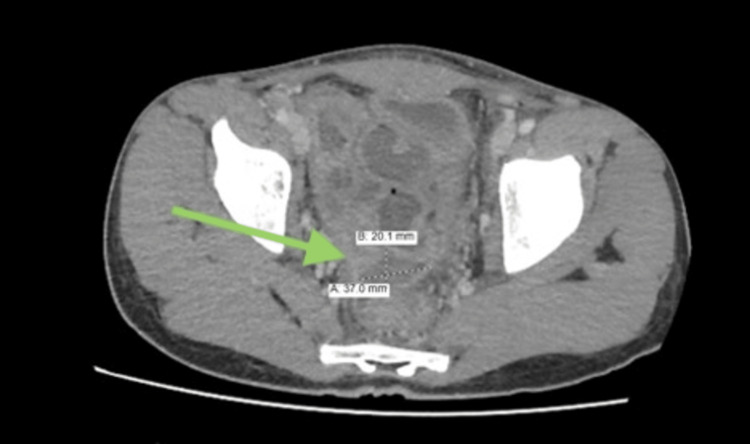
An axial CT image was obtained on readmission showing interval development of a multiloculated presacral fluid collection measuring approximately 15 mL, indicating intra-abdominal abscess formation.

**Figure 3:**
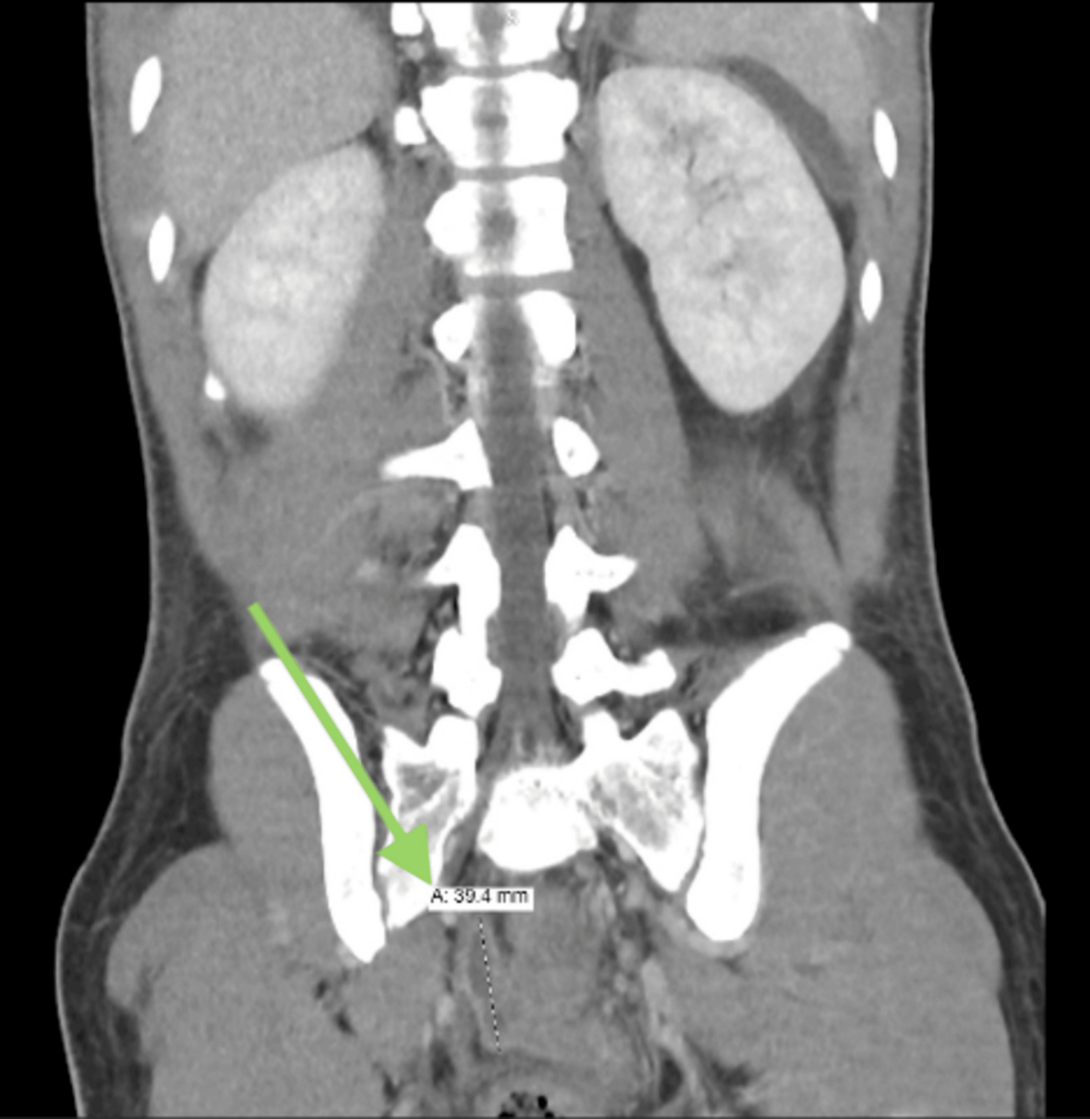
Follow-up CT image displaying progressive thickening and enhancement of the peritoneum with increased ascitic fluid, consistent with ongoing peritonitis.

The patient demonstrated initial improvement postoperatively and was discharged on the fourth day with oral ciprofloxacin and metronidazole. However, he returned two days later with recurrent abdominal pain. Repeat CT imaging revealed a presacral multiloculated fluid collection, consistent with ongoing peritonitis (Figures 2, 3). Culture results from the initial intraoperative swab grew extended-spectrum beta-lactamases (ESBL)-producing *Escherichia coli* and *Pseudomonas aeruginosa*, prompting an upgrade to meropenem for a 14-day course of antibiotics.

**Figure 4:**
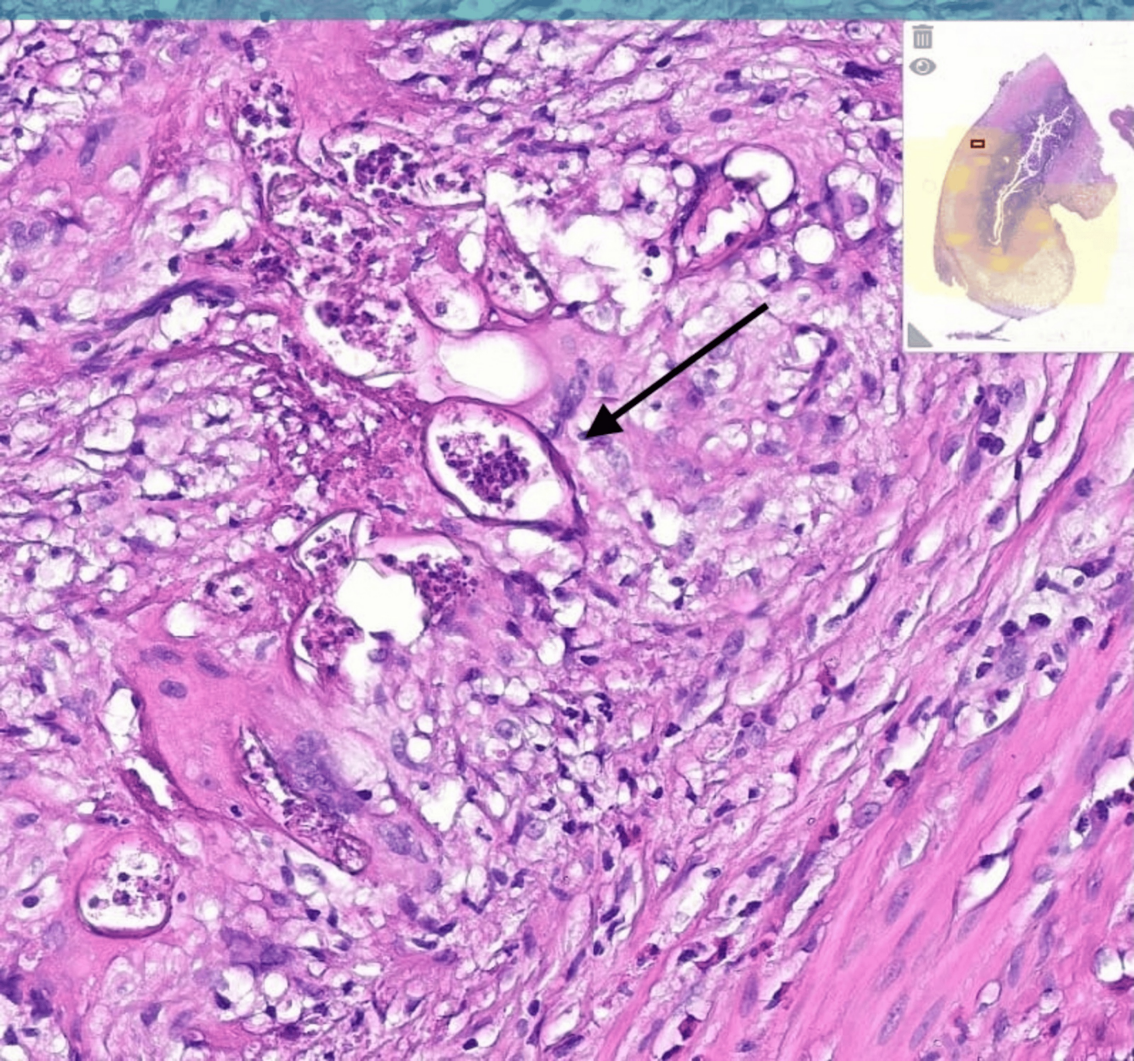
Histopathological section of the appendix showing multiple Schistosoma ova embedded within the appendiceal wall, surrounded by granulomatous inflammation and eosinophilic infiltrates, histopathological section of the appendix showing multiple Schistosoma ova embedded within the appendiceal wall, surrounded by granulomatous inflammation and eosinophilic infiltrates , stain (Hematoxylin and Eosin), and magnification level (×100).

Histopathological analysis of the excised appendix revealed schistosomal ova, confirming the diagnosis of appendicular schistosomiasis. The patient subsequently reported frequent exposure to freshwater lakes during his time in Yemen, likely the source of infection (Figure 4).

The patient was treated with praziquantel in addition to antibiotics and showed significant clinical improvement. At the one-month follow-up, the patient was doing well, with a clean but not fully healed laparotomy wound. He was advised to continue local wound care and was scheduled for outpatient follow-up.

## Discussion

Schistosomal appendicitis is an uncommon manifestation of schistosomiasis, with its incidence being particularly low compared to other gastrointestinal complications of the disease [3]. The exact pathophysiology remains unclear, but the mechanical obstruction of the appendiceal lumen due to egg deposition and subsequent inflammatory response is believed to contribute significantly to the condition [9]. The resulting tissue damage may lead to necrosis and perforation in rare cases, culminating in peritonitis [10].

The clinical presentation of schistosomal appendicitis is often indistinguishable from that of typical acute appendicitis, with symptoms such as right lower quadrant abdominal pain, nausea, vomiting, and fever [11]. As a result, preoperative diagnosis is rarely established, and most cases are only identified postoperatively through histopathological examination of the resected appendix [12]. A study in Senegal reported two cases where appendicitis was caused by *Schistosoma* species, further supporting the importance of considering parasitic infections in appendiceal pathology [9].

Management of schistosomal appendicitis involves a combination of surgical intervention and antiparasitic therapy. Appendectomy remains the definitive treatment for acute cases, while praziquantel is the drug of choice for eradicating the underlying schistosomal infection [6]. Some studies suggest that post-surgical antiparasitic treatment may prevent disease recurrence and associated complications [13].

This case emphasizes the need for clinicians to maintain a high index of suspicion for parasitic infections in patients from endemic regions presenting with acute abdomen. Routine histopathological evaluation of appendectomy specimens can aid in identifying unexpected causes of appendicitis, such as schistosomiasis, facilitating appropriate postoperative management, and preventing disease recurrence [8].

## Conclusions

This case highlights a rare presentation of appendicular schistosomiasis resulting in peritonitis. It underscores the importance of considering parasitic infections such as schistosomiasis in the differential diagnosis of acute appendicitis, particularly in patients from endemic regions or with relevant travel histories. Histopathological examination of the resected appendix played a critical role in confirming the diagnosis. In endemic areas, routine stool ova and parasite (O&P) testing or serologic screening pre-operatively could aid in earlier recognition and guide appropriate antiparasitic management.
